# Women Who Practiced Medicine in Ancient Times

**Published:** 1861-03

**Authors:** 


### Editors' Table.

Women who Practiced Medicine in Ancient Times.—In our brief analysis of the major part of Dr. Rouyer's treatise,* we intimated our intention to notice, at a future day, the final chapter, which bore for title, "*History of Women who have Practiced Medicine.*" Our remarks on the subject, at this time, will be chiefly based upon said chapter of the work in question.

From the most remote antiquity, or, more correctly speaking, from the earliest period of historic record, and anterior to it, again, of mythical story, women have been engaged in the practice of midwifery, and even other branches of the healing art. Homer, Hippocrates, and Galen may be quoted to this effect. The popular belief interwoven with their mythology, shows the influence the Greeks attached to female interposition in the person of their goddesses, as aiding or superintending the labor of parturient females. Thus Juno, who occupies the first place on the list, was, under the names of Lucina or Lucinia, invoked by pregnant and parturient women. It is true that infants came into the world under the protection also of Jupiter *Diespiter*. After their birth the goddess *Cunina* watched over their cradle, and at a later period the *Carmentes* presided over their future destiny. During pregnancy the goddess *Genia Mana* was invoked to prevent the child being born feeble or deformed.

Coming to the heroic period, we find the names of a number of women connected with the healing art. The centaur Chiron, who was the preceptor of so many heroes and physicians, had a daughter Hippo, who was versed in medicine and pharmacy, which she afterward taught to Eolus; she was also skilled in the art of divination, as was her sister Ocyrsæ, who predicted the future greatness of Æsculapius. Epione, a daughter of Hercules, who had been himself one of the pupils of Chiron, was famed for her medical knowledge, which she inherited from her father. The women who figured during this age as adepts in the art of healing, had also the reputation of being given to sorcery. Among these we meet with Hecate, wife of Ætes, King of Colchis, and mother of Circe, Medea, and Angitia, who were said to have great experience in medicine. Hecate was well acquainted with the preparation of poisons, a knowledge which she turned to fatal account by poisoning her father, in order to secure for herself the succession to the throne. She discovered the toxical properties of aconite. Medea found antidotes to poisons, and gave proofs of supernatural power by turning men into lions, wolves, and swine. She was represented to be acquainted with everything relating to the art of medicine. She was very benevolent in saving men from threatened dangers, and

* Etudes Médicales sur l'Ancienne Rome.

acted the part of a dutiful wife in dressing the wounds of her husband, Jason, who was another of the pupils of Chiron. She incurred the imputation of boiling men to death—a prejudice against her which arose from her causing aged persons, and among others her father, to be immersed in the warm bath, from which they came out renovated, and, as was said, rejuvenated. In a more figurative sense, she restored to old men their youth by imparting to their gray hair a black tint. Circe, celebrated by Homer and Virgil, imitated the bad example of her mother, Hecate, by poisoning her husband, King of the Sarmatians, in order that she might rule alone. She was obliged, however, to fly the kingdom for this enormity, and take refuge in Italy. The fiction of the transformation into swine of those who drank from the cup offered by Circe, is generally received as typical of those who make beasts of themselves by getting drunk on alcoholic liquors. Many of the female members of the family of Æsculapius obtained credit for attainments in medicine. It is probable that the various names given to the daughters of the god of medicine, as well as to his wife Epione, were used in a figurative sense, to imply health, cure, and strength. Medals were struck and statues raised to their memory by their grateful patients. Helen, the fair and frail wife of Menelaus, whose elopement with Paris cost Troy so dear, and enriched literature for all time with the Iliad, had some knowledge of medicine. Helenium was named after her; and she was said to be acquainted with the exhilarating properties of the nepenthes. Œnone, the unhappy wife of Paris, was also acquainted with plants; but she refused to apply the remedies of which she alone was the possessor to treat the wounds of Paris; although, woman-like, she afterward, on his death, killed herself in despair. Machaon, who played soldier as well as surgeon at the siege of Troy, was glad to avail himself of the services of Acamede and Hecamede, to dress his wound.

Escaping from the gray morn of the heroic age, with its dimly seen outlines of humanity, we come to the clear day of history. At Athens men were engaged in the practice of midwifery, but in a limited degree, and only casually. This circumstance explains, according to some commentators, why we meet in the books of Hippocrates such scant notices of obstetrics. To this statement it has been objected, that women were prohibited from the practice of medicine by an express law of the State. But if such a law had ever existed it was not allowed to be long in force, and was repealed in compliment to Agnodice, who studied midwifery under a certain Herophilus, or Hierophilus. After her novitiate had expired, she determined to don the male attire, in order that she might not be subjected to the penalty of the existing prohibitory law against females practicing medicine. If any of her patients was backward in receiving her services she immediately revealed her sex, and took charge of the case without any further demur. The great popularity of Agnodice elicited the envy of the physicians, who accused her of corrupt practices and of being a debauchee, to procure whose visits the women would simulate diseases. When cited to appear before the Areopagus, she withdrew her tunic and convinced the assembly that she was a woman. Many of her sex now bestirred themselves in her behalf, and made a brief and pithy address to the Areopagites which was so effective as to induce them to repeal the old law.

Plato furnishes tolerably extensive information respecting the duties of midwives, in the dialogue between Socrates and Thectêtes, in which the former asks his interlocutor: "Can you be ignorant that I am the son of a skillful and renowned midwife, Phenareta?" On the other replying that he has heard tell of the circumstance, Socrates continues: "Call well to mind all that relates to midwives. You know well that none of them can take a part in delivering women so long as they themselves continue to be mothers, and that they only follow this business after they have become incapable of conception themselves." "The practice is attributed to Diana, at least so it is said, because, without being a mother herself, she presides over child-births. She would not confide this employment to barren women; human nature being unequal to the practice of an art of which they could have no experience." Another remark of Socrates in this dialogue throws light on the ethics of the Greeks, in regard to abortion procured with the consent of the pregnant woman. In speaking of the functions of midwives, he says: "They are able, even, by remedies and enchantments, to bring on the pains of child-birth, as well as to moderate them, or to bring on abortion of the child, when the mother is determined to get clear of it." The great philosopher then adverts to midwives being the agents in negotiating marriages, on account of their being able to tell accurately what man and what woman it is proper to unite together, in order that they may have the finest children. "Well, be assured," he continues, "they are prouder of this talent than even of their skill in cutting the umbilical cord." Corrupt pretenders and intruders having usurped the agency business of the regular midwives, these latter, out of respect for themselves, declined matrimonial commissions, as not strictly pertaining to their legitimate office.

The Greek midwife was called on to give her attention to women during pregnancy, and during and after their confinement; she was also expected to furnish them with the necessary medicines and to indicate to them the hygienic rules to be observed during their pregnancy, and she took part, also, in certain ceremonies which were performed after child-birth. Uterine disorders, and all the illusory practices for treating sterility, were also among the attributes of this class. Moschion, one of the first authors who has written expressly on the diseases of women, specifies the requisite qualities in a midwife. She is to be preferred, "who has gone through literary studies, who is intelligent and has a good memory; she must be studious, alert, strong, free from impurity or disease, and be given neither to anger nor to broils. She must be, besides, compassionate, sober, modest, observing, quiet, prudent, and not a miser." No imperfect list of qualifications these, either for a woman or man practitioner of the art of midwifery. Moschion adds another condition, relating to their dress. She must be in male attire. "*Viriliter cuncta sit.*" In another place, Moschion, in reply to the question: How many assistants must the midwife have? says three; one on each side of the woman in labor, and the third behind her, so as to prevent her from turning herself in the wrong direction during the pains of labor. These assistants might, after a sufficient time, and knowledge of the practice of midwifery, become, themselves, midwives—

*obstetrices.* Aristotle, in his work on the history of animals, speaks of the necessary qualifications for a midwife, in order that she may be prepared to remedy the various complications that may present themselves during labor, and to cut the umbilical cord, an operation to which he attaches great importance.

The number of Greek midwives whose names have come down to us is small. We may cite these of Agnodice, already mentioned, Olympias of Thebes, Lais, Elephantis, Sotira, and Salpe. It is not yet determined by the critics whether Lais the midwife was, also, Lais the courtezan, who gave her favors to the philosophers Aristippus and Diogenes, and to Demosthenes the orator. Elephantis wrote on abortives and cosmetics, but neither these works, nor the very licentious ones of which she was the author, and which were read with such delight by Tiberius at Capri, have reached us. To the list already given we may add the names of Phenareta, the mother of Socrates, Antiochis, and Philista, sister of the philosopher Pyrrho, and, more celebrated than all, Aspasia. The question of the identity of this Aspasia with the mistress of Pericles and the favorite of Socrates, is generally replied to in the negative. Among the varied accomplishments of Aspasia of Miletus, specified by Lucian, in arts, philosophy, and science, no mention is made of her knowledge of medicine, nor of having written on midwifery. Ætius has preserved the titles of the fragments of the other Aspasia, on nine different subjects relating to the diseases of women, and displacements of the uterus, and attention after abortion. Of a still higher rank than any hitherto named by us, in the list of female practitioners of midwifery, is the celebrated Cleopatra, Queen of Egypt, who led Mark Antony into those luxurious indulgences and vagaries which probably cost him the rule of the Roman Empire, and allowed the ascendency of his more fortunate rival Octavius, afterward Augustus Cæsar.

The Romans, in adopting Greek manners and social usages, did not neglect to employ midwives. The first notice of this class of women in Rome is found in Plautus,* who speaks of a midwife complaining that she was not paid enough. *Tum obstetrix expostulavit mecum parum missam sibi.*—Terence, who came after Plautus, speaks also of midwives, in the comedy of Andria, in which reference is made to a midwife given to drink, and who on this account was not worthy of confidence. There were in Rome regular midwives—*obstetrices*, and others called *adstetrices*, a title which would seem to imply that they were aids to the first-mentioned class. There was yet another class called *sagæ*, whence the French *sage*-femme. The office of a *saga* was not well defined; the word was sometimes used to designate a sorceress or a magician, a go-between, a perfumer, and a midwife; but the word was never used in good part, and it was generally applied to those women who exercised those different vocations.

In reference to the *obstetrices* and their functions, we learn that a happy conception was prepared by the attentions of the midwife—*ab initio oriendum est.* Then, again, the growth of the fœtus was to be facilitated by an appropriate regimen. "*Brassicum comedunt ad fœtûs incrementum; ab-*

* Miles Gloriosus.

*stinent a sale et aquâ frigidâ. Lecticis vehumtur et equabus gravibus inequitant, etc.*" While no objection need be made as a matter of taste to the latter part of these directions, that pregnant ladies shall be carried on a litter, and ride on mares with foal, many would demur to abstinence from salt and cold water, and to the eating of cabbage as a means of aiding the growth of the child *in utero*. We shall not repeat the names of the various divinities appealed to on the several periods just preceding, during, and after labor, in order to render safe and easy the several changes at these times. The Roman midwife, as we learn from Pliny, made use of quite a variety of medicaments when labor was coming on, in order to prepare the genital passages for the easy expulsion of the fœtus. Of the superstitious observances required on the occasion, it will be sufficient for us to notice that one which forbade any person in the house to have his legs crossed, or his fingers interlocked, for if this were allowed the labor would be suspended.

On the midwife devolved the duty of washing the infant and giving it the earliest attention. Different liquids were used in different countries. At Athens, water was employed, and afterward oil. At Sparta, the child was washed with wine; and among some others of the people of Greece, by a refinement bordering on the sentimental, dew was had recourse to. The Cimbri employed snow for the purpose. Sometimes the ablutions were made in vases of a particular fashion. At Sparta the children were placed on a shield, with a lance near it, in order to show that they were devoted to the defense of the State. In the family of Julius Cæsar, the vase appropriated to the purpose was the back or shield of a turtle. When the father himself washed the child, this act was regarded as a proof of extreme love for his offspring. The functions of the midwife terminated after five days; and she then gave place to the nurse who was to suckle the child and to watch over it for a long period. In case of sickness the midwife was called in. For the most part the medication consisted of certain superstitious practices, such as applying amulets to the body of the child; and in order to give efficacy to these fashions, sacrifices were offered up to Juno-Lucina, and to Castor and Pollux.

On the third day after the birth of the child among the Romans, and the fifth day among the Greeks, a crown was suspended over the door of the house. At Athens an olive crown was so placed to announce the birth of a boy, and a woollen one for that of a girl. At Rome the crowns were made of laurel, ivy, marsh celery, and aromatic herbs. On the eighth day from the birth of a girl and in the ninth in the case of a boy, (*lustrici dies,*) a name was given to the child; and care was taken that this should be a decent one, for at a later period the bearer of a base or impure name was punished. Finally, on the third day from the naming of the child, it was formally registered by the inscription of its name, prenames, and surnames, and to which were added the date of its birth and the names of the consuls who were in office for that year.

The names of some Roman widwives have reached us: Fabulla Lybica or Livia is quoted by Galen, and Victoria, Salviana or Salvina, and Leoparda are mentioned by Priscian. Scubonius Largus bought of another of this class, named Africana, the knowledge of a secret remedy for colic.

We may believe that, in addition to recognized midwives in Rome, there were women who were engaged in the treatment of diseases, and hence they were called *medicæ*, although the details on this point are not very explicit, if we except what St. Jerome says of a Roman lady, called Fabiola, who lived in the fourth century after Christ, and who founded a hospital in which she herself took charge of the sick. The pathology of the Roman times bore hard on the menstrual fluid, as something extremely damaging to all persons and things in the presence of the female during its flow. Wine became sour, plants sterile, the gloss of purple garments and the polish of mirrors were tarnished, and both steel and iron were corroded, bees were chased from their hives, mares miscarried, the edge of a razor was dulled in the hands of the barber. But, on the other hand, this diffusive influence sometimes did good, as, for example, if a woman who was menstruating walked round a field, all the grubs and destructive insects fell to the ground. Lais and Elephantis have written on this point, in very different strains; for while one asserts that fecundity is insured by taking of the ashes of burnt menstrual fluid, the other insists that sterility would certainly follow the use of this article. The safest course advised by M. Rouyer is not to believe either of these lady doctors.

What we know of the Jewish midwives is derived from the Bible, as, for instance, in the accounts of the child-bearing of Rachel and Thamar. The same rich history tells us of the conduct of the Egyptian midwives in their declining to obey the cruel mandate of Pharaoh, that they should destroy all the male children of the Jewish women, of whom it would seem they had the charge on such an occasion. The Arabians had two orders of female practitioners, viz., *mulier medica*, regular doctors, and *mulier obstetrix*, midwife.

